# Surveillance and Preparedness for Ebola Virus Disease — New York City, 2014

**Published:** 2014-10-17

**Authors:** Isaac Benowitz, Joel Ackelsberg, Sharon E. Balter, Jennifer C. Baumgartner, Catherine Dentinger, Anne D. Fine, Scott A. Harper, Lucretia E. Jones, Fabienne Laraque, Ellen H. Lee, Giselle Merizalde, Celia Quinn, Sally Slavinski, Ann I. Winters, Don Weiss, Kari A. Yacisin, Jay K. Varma, Marcelle C. Layton

**Affiliations:** 1 Epidemic Intelligence Service, CDC; 2 Bureau of Communicable Disease, Department of Health and Mental Hygiene, New York, NY; 3 Career Epidemiology Field Officer, Office of Public Health Preparedness and Response, CDC; 4 Office of Emergency Planning and Response, Department of Health and Mental Hygiene, New York, NY; 5 Division of Epidemiology, Department of Health and Mental Hygiene, New York, NY

In July 2014, as the Ebola virus disease (Ebola) epidemic expanded in Guinea, Liberia, and Sierra Leone, an air traveler brought Ebola to Nigeria and two American health care workers in West Africa were diagnosed with Ebola and later medically evacuated to a U.S. hospital. New York City (NYC) is a frequent port of entry for travelers from West Africa, a home to communities of West African immigrants who travel back to their home countries, and a home to health care workers who travel to West Africa to treat Ebola patients. Ongoing transmission of *Ebolavirus* in West Africa could result in an infected person arriving in NYC. The announcement on September 30 of an Ebola case diagnosed in Texas in a person who had recently arrived from an Ebola-affected country further reinforced the need in NYC for local preparedness for Ebola.

To ensure that NYC is prepared to manage Ebola cases and prevent disease transmission, the NYC Department of Health and Mental Hygiene (DOHMH), in close coordination with local hospitals and clinicians, nongovernmental organizations and community groups, and city, state, and federal agencies, established systems around Ebola surveillance and management of suspected cases and contacts, and built upon existing general protocols for early recognition and management of persons with a viral hemorrhagic fever. Objectives included rapidly identifying Ebola patients in health care settings, implementing infection control precautions, and transporting ill persons to hospitals via emergency medical services, including persons arriving on international flights into John F. Kennedy International Airport. Enhanced planning began immediately after a CDC alert about Ebola on July 28, 2014. Reporting criteria and infection control guidance were developed in collaboration with local hospitals and sent to hospitals and clinicians via an electronic health alert system on August 11. Information also was shared on three citywide conference calls and in oral presentations to target audiences (1). DOHMH developed Ebola-specific data collection forms and triage protocols and trained staff to handle calls.

The guidance instructed clinicians to call DOHMH immediately after identifying any patient meeting the CDC definition for a person under investigation (PUI): a person who traveled to an Ebola-affected area within 21 days of onset of symptoms and had fever >101.5° F [38.5° C] and compatible symptoms such as severe headache, muscle pain, vomiting, diarrhea, abdominal pain, or unexplained bleeding (Figure) (2,3).* The guidance provided a link to the CDC website for information on the current list of affected areas (4). DOHMH also assisted area hospitals in planning for isolation and management of PUIs or confirmed Ebola patients. DOHMH distributed posters for health care facilities to post in emergency departments to encourage patients to report recent travel history to an Ebola-affected country upon arrival.† DOHMH medical epidemiologists were available at all hours to respond to clinician and hospital questions about PUIs or other persons suspected of having Ebola, using guidance largely consistent with CDC’s risk categories. Under the system, patients with high-risk or low-risk exposure to Ebola would be transferred to another hospital if there was concern about the ability of the reporting hospital to manage the patient; Ebola testing, if indicated and after consultation with CDC, could be performed at DOHMH with confirmatory testing at CDC. Patients should also undergo evaluation for alternate diagnoses. The protocol included consideration of laboratory studies such as complete blood count, coagulation studies, liver function tests, and malaria testing, to assist in determining the need for Ebola testing. Patients not needing hospitalization could remain isolated at home, with daily monitoring by telephone by medical epidemiologists until the patient’s symptoms improved such that Ebola was no longer of concern, or until worsening or persistent symptoms prompted repeat evaluation for Ebola or an alternate diagnosis.

As of October 6, 2014, DOHMH had received inquiries from health care providers about 88 patients: 49 (56%) had not been in an affected area in the 21 days before symptom onset, and 28 (32%) met travel criteria but not clinical criteria. Of the 11 (12%) who met PUI criteria, none had any high-risk or low-risk exposure factors. One was tested for Ebola, and the test result was negative. Alternate diagnoses included malaria (8 patients) and typhoid fever (one patient); two others had no clear diagnosis. Two patients were discharged home while febrile and remained isolated at home for several days; all of the patients recovered. Some patients had potential delays in diagnosis because of hesitancy by health care providers to examine patients or by laboratory workers to handle specimens.

These experiences demonstrated the feasibility of rapidly implementing enhanced surveillance for Ebola-like illness. A second electronic health alert, sent on September 3, highlighted the need to obtain a full travel history from febrile patients and consider alternate diagnoses particularly in patients with no known exposure and emphasized that no added precautions are needed to perform laboratory studies on those patients (5,6).

NYC has previously faced threats to human health from outbreaks occurring overseas, including from plague, severe acute respiratory syndrome, measles, novel influenza strains with pandemic potential, and more recently Middle East respiratory syndrome (7). The need to take a full travel history on any patient presenting with a febrile illness, and to remain aware of current overseas outbreaks, is not new. Provider awareness and media attention peak when an emerging threat is first recognized, but such threats can persist for months. The recent diagnosis of Ebola in a person in the United States who had traveled from an affected area underscores the need for health departments to prepare to rapidly respond to imported cases. It is challenging for health officials and health care providers to stay vigilant for high-consequence but low-likelihood events and to maintain a high level of preparedness for managing such events safely. Critical elements highlighted in this report include the development of clear reporting criteria, building and maintaining relationships and preparedness capacity in the local health care system, and rapid, frequent and responsive communication with the health care community and the public to identify and address concerns.

## Figures and Tables

**FIGURE:**
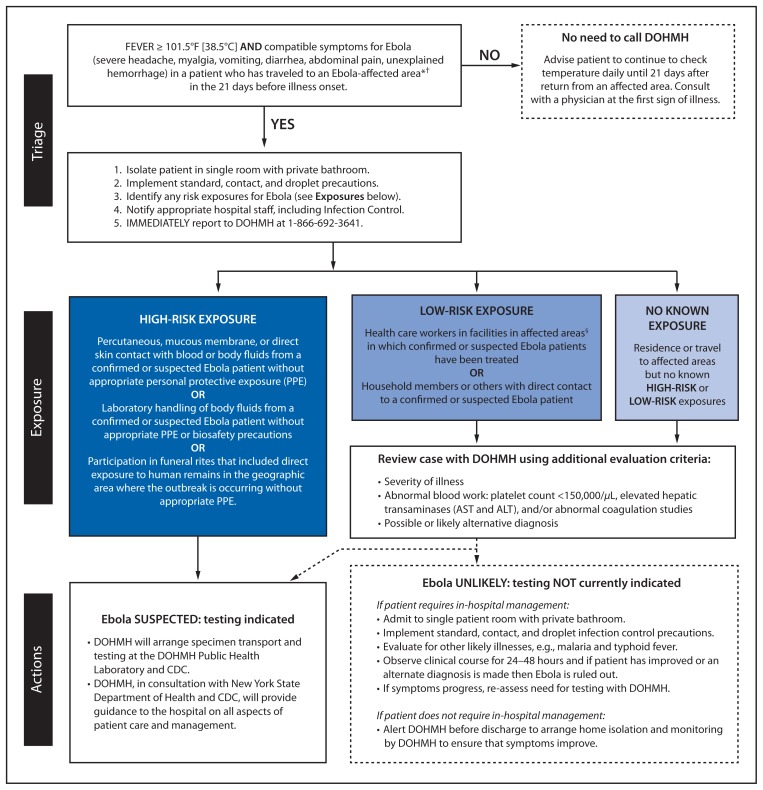
Ebola virus disease (Ebola) evaluation algorithm — New York City Department of Health and Mental Hygiene (DOHMH), September 3, 2014 **Abbreviations:** AST = aspartate aminotransferase; ALT = alanine aminotransferase. * The current list of affected areas is available at http://www.cdc.gov/vhf/ebola/outbreaks/2014-west-africa/distribution-map.html. ^†^ On October 9, DOHMH revised its reporting criteria to include fever or other compatible symptoms. ^§^ In the CDC algorithm, health care workers using appropriate PPE in facilities with Ebola patients are classified as having no known exposure, but, according to DOHMH guidance, if they develop fever and compatible symptoms in the 21 days after residence in or travel to an Ebola-affected area, they are considered to have had low-risk exposure.

## Footnotes

* On October 9, DOHMH revised its reporting criteria to include fever or other compatible symptoms.

† A sample poster is available at http://www.nyc.gov/html/doh/downloads/pdf/em/hospital-signs-tabloid.pdf.
